# Refractory lympho-epithelial carcinoma of the nasopharynx: a case report illustrating a protracted clinical course

**DOI:** 10.1186/1758-3284-1-18

**Published:** 2009-06-15

**Authors:** Frederick Y Wu, Eddy S Yang, Christopher D Willey, Kim Ely, Gaelyn Garrett, Anthony J Cmelak

**Affiliations:** 1 Department of Radiation Oncology, Vanderbilt University School of Medicine, Nashville, TN, USA; 2 Department of Radiation Oncology, University of California at San Francisco, San Francisco, CA, USA; 3 Department of Pathology, Vanderbilt University School of Medicine, Nashville, TN, USA; 4 Department of Otolaryngology, Voice Center, Vanderbilt University School of Medicine, Nashville, TN, USA

## Abstract

Nasopharyngeal carcinoma is an uncommon cancer in North America. Its clinical course is typified by locally advanced disease at diagnosis and has a high propensity for both regional and distant spread. It is, therefore, typically treated with a combination of radiation and chemotherapy. This report describes our 10-year clinical and radiological findings in a 48-year-old Vietnamese male patient with locally-advanced T4N1M0 lympho-epithelial carcinoma of the nasopharynx. Despite a long remission period after his initial course of aggressive chemoradiation, his tumor recurred locally after 4 years. Thereafter, throughout a period of over 10 years, he has been treated with multiple courses of re-irradiation and three different trials of chemotherapy. He was ultimately provided with over 30 months of progression-free tumor control with the epidermal growth factor receptor (EGFR)-inhibitor cetuximab. This case illustrates the commonly protracted course of this disease and its responsiveness to multiple treatment modalities.

## Introduction

Nasopharyngeal carcinoma (NPC) is a squamous cell carcinoma that occurs in the epithelial lining of the nasopharynx. In the United States and Western Europe, nasopharyngeal carcinoma is a relatively rare. It is more common among Southern Chinese, Southeast Asian, Northern African, and Eskimo populations [1]. Among head and neck cancers, NPC has the highest propensity for developing distant metastases. Histologies of NPC range from well-differentiated squamous carcinoma (WHO type I), to non-keratinizing squamous carcinoma (WHO type II), to lympho-epithelial carcinoma (WHO type III), where non-keratinizing squamous carcinoma cells are mixed with numerous benign lymphocytes. Type III cancers are regarded as having the highest response to treatment, but also the greatest propensity for developing distant micrometastatic spread. Due to the anatomical location of the disease, NPC is typically not treated with surgical resection; rather concurrent chemoradiotherapy is the preferred approach. Cisplatin with radiation followed by adjuvant chemotherapy has been reported to be more effective in patients with NPC than with radiation alone [2,3], and this approach has now been adopted as current standard of care. Despite this combined approach, 31% of patients will develop recurrent disease[2].

Cetuximab, a recombinant human/mouse chimeric monoclonal antibody, binds specifically to the epidermal growth factor receptor (EGFR, HER1, c-ErbB-1) and competitively inhibits the binding of epidermal growth factor (EGF). This results in inhibition of cell growth, induction of apoptosis, and decreased matrix metalloproteinase and vascular endothelial growth factor production [4]. This drug has proven clinical benefit as a single agent in patients with recurrent or metastatic squamous cell carcinoma of head and neck [5,6]. Additionally, a phase III trial was published showing improved local control and overall survival in patients treated with cetuximab plus radiation compared to radiation alone [7].

The following case report describes a Vietnamese male with a 10-year clinical course of lympho-epithelial carcinoma of the nasopharynx who failed multiple therapies involving both chemotherapy and radiation, but was able to achieve stable disease for over 30 months with EGFR inhibition.

## Case report

**Figure 1:**
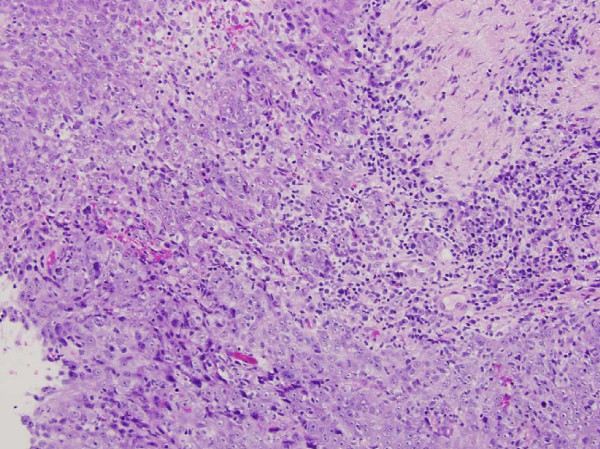
**Pathology from original biopsy**. Note the characteristic proliferation of cells arranged in a syncytial pattern as well as the vesicular nuclei with prominent nucleoli and areas of keratinization.

**Figure 2:**
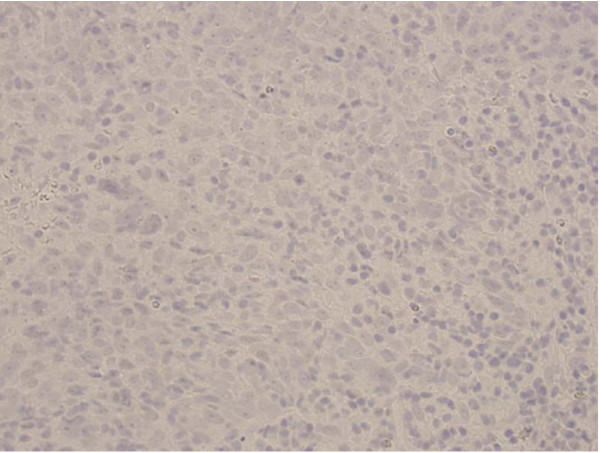
**Negative EBV latent membrane staining of biopsy specimen**. This test, however, is not as sensitive or specific as the EBER test which became available after this patient was diagnosed.

**Figure 3:**
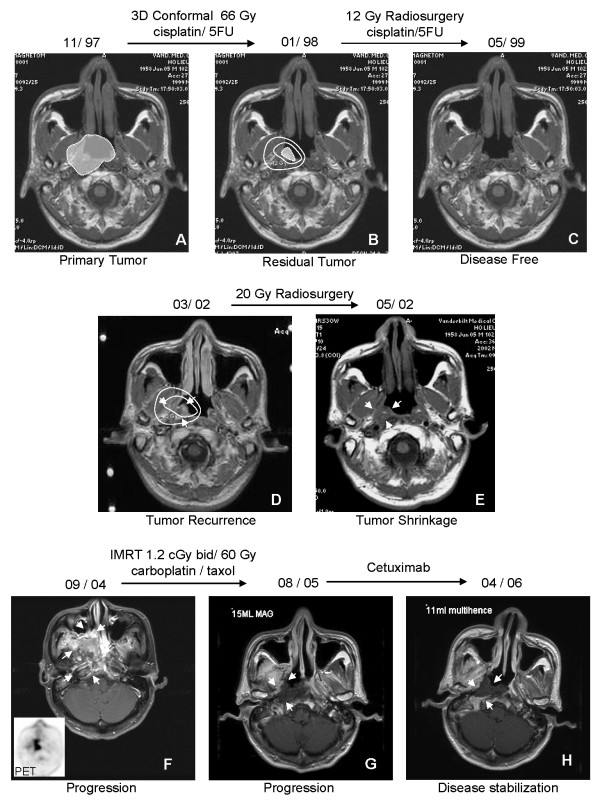
**Multiple MRI scans of the nasopharynx listed in chronological order showing the progression of disease after multiple treatments**. In Part A and B, the extent of the tumor was indicated with the white enclosed areas. In Part C to H, boundaries of the tumor are indicated (arrows). Dosimetries of the radiosurgery are indicated in Part B and D (concentric curved circles). Part F contained FDG-PET scan of the same period in the same nasopharyngeal plane as the MRI.

In October 1997, a 39 year old immigrant from Vietnam presented with right-sided headache, otalgia, and tinnitus over the previous month. He was discharged with clarithromycin for suspected otitis media. One month later, he returned with severe right otalgia which now radiated to the vertex of his skull. He re-presented with continued symptoms. Endoscopic exam revealed a large mass emanating from the nasopharynx. Biopsy was performed, with surgical pathology revealing poorly-differentiated invasive lympho-epithelial nasopharyngeal carcinoma (Figure 1). Stains for the Epstein Barr Virus (EBV) latent membrane protein were negative in the neoplastic cells (Figure 2). This test, however, is not as sensitive or specific as the EBER test which was not available at the time. Neck MRI confirmed a large 4.0 × 4.7 × 5.4 cm mass emanating from the nasopharynx and invading the orifice of the right Eustachian tube. A necrotic cervical lymph node in the right spinal accessory chain was pathologically enlarged at 1.3 cm and was suspicious for metastatic disease (Figure 3, part A). Chest x-ray and bone scan were negative, and liver function tests were within normal limits. He was staged (AJCC staging 1997) with stage T4N1M0 nasopharyngeal carcinoma.

The patient was referred for chemoradiation treatment, and in 11/1997, he began concurrent cisplatin (100 mg/m^2^) with concomitant 3D conformal radiation therapy consisting of 66 Gy in 33 fractions of 2.0 Gy. During radiotherapy, he developed severe mucositis, dysphagia and odynophagia requiring temporary PEG tube placement. Post-treatment MRI in 1/1998 showed marked interval decrease in size of the right nasopharyngeal tumor, which now measured approximately 2.2 × 1.3 cm. Repeat MRI in 2/1998 showed a small quantity of residual fullness in the right nasopharynx (Figure 3, part B). The patient, therefore, subsequently received a stereotactic radiosurgical boost (1200 cGy in one fraction) to the residual abnormality as well as three cycles of adjuvant 75 mg/m^2 ^cisplatin and 5-fluorouracil 1 g/m^2 ^(d1-5) every 21 days. The patient did not tolerate this regimen well with the development of leukocytopenia and grade IV mucositis, which necessitated a 50% reduction of cisplatin and 5-FU dose on cycles 2 and 3.

By 6/1998, however, the patient had recovered well and showed no evidence of disease on physical and endoscopic examination. MRI 7/1998 showed no obvious disease, only slight soft tissue asymmetry within the nasopharynx. He remained disease-free on frequent follow-up examinations and MRI until 5/1999, when he presented with severe, crippling, lancinating headaches that initiated at the occiput and radiated to his right eye. This was associated with severe fatigue, weakness, and gait instability. An MRI at that time showed no evidence of disease (Figure 3, part C). Therefore, the patient was treated conservatively with Gabapentin for presumed neuropathic pain that provided good symptomatic relief. He remained clinically NED for over 2 years.

**Figure 4:**
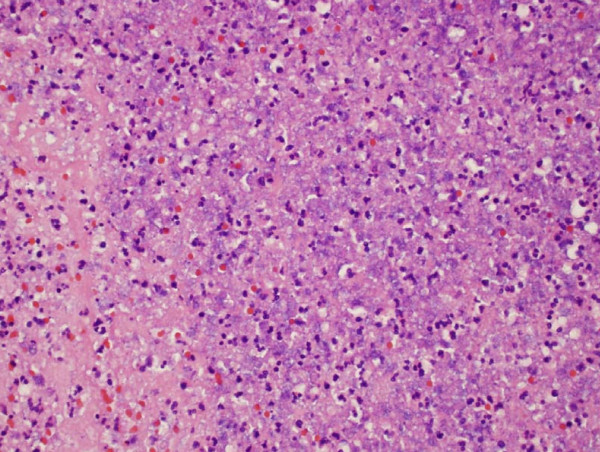
**Pathology from recurrence in 1/2002**. Note the increased tumor necrosis compared to 1997.

However, in 11/2001, almost 4 years after the treatment of his initial disease, repeat neck CT showed an interval increase in the asymmetry seen within the soft tissue of his right nasopharynx. Flexible fiberoptic endoscopy revealed a right-sided 1 cm nasopharyngeal ulcer in the fossa of Rosenmüller. The patient was scheduled for a biopsy of the lesion, but the patient missed the subsequent appointment. In 1/2002, he did return to the clinic but reported symptoms of dizziness, fullness of his ears, fatigue, and occasional headache. He was unable to tolerate solid food. Repeat MRI showed persistent fullness within the right fossa of Rosenmüller. However, the size of the soft tissue abnormality remained unchanged compared with the prior CT. On 1/29/2002, nasal endoscopy with nasopharyngeal biopsy revealed poorly-differentiated nasopharyngeal carcinoma identical to his initial tumor histology (Figure 4). Both MRI and CT in 3/2002 were used to outline the area of recurrence (Figure 3, part D) and he subsequently underwent LINAC-based stereotactic radiosurgery as salvage treatment, delivering 2000 cGy in a single fraction to the periphery of the tumor. The treatment was well tolerated with no major complications.

Repeat MRI in 5/2002 showed a significant decrease in the right-sided nasopharyngeal fullness, but some right parapharyngeal edema remained (Figure 3, part E). The patient noted no significant detriment in his swallowing or speech. Almost one year later, in 4/2003, he reported numbness over the right trigeminal (V1-V3) dermatomes as well as right ear hearing loss and epistaxis. Unfortunately, the patient missed several of his follow-up and MRI appointments.

When he returned to clinic in 9/2004, his MRI showed significant progression of disease. There was a 3 cm mass that extended through the right skull base and foramen ovale. Additionally, tumor was seen in the right pterygopalatine fossa with destruction of the posterior maxillary sinus extending to the posterior aspect of the right orbit. The tumor also extended laterally to the infratemporal fossa, invading the medial and lateral pterygoid muscles. There was tumor invasion into the longus coli muscle and the perivertebral space with involvement of the jugular tubercle and right jugular foramen. The mass extended inferiorly to the right tonsils, and there were multiple pathologically-enlarged right submandibular and submental lymph nodes (Figure 3, part F).

The patient subsequently received weekly chemotherapy with carboplatin and paclitaxel in 9/2004 for palliation of symptoms. During treatment, patient reported trismus and right ear/jaw pain, and he was started on amoxicillin for a possible infectious process. Repeat MRI of 12/2004 showed slight interval decrease in the size of the skull base component of the nasopharyngeal mass when compared to the 9/2004 study. However, the tumor showed evidence of perineural spread and direct invasion into the skull base and right cavernous sinus as well as into the pterygopalatine fossa and right muscles of mastication.

He continued on weekly carboplatin/paclitaxel. Repeat MRI 2/2005 showed minimal decrease in the size of the nasopharyngeal portion of the mass. Moreover, the patient became leukopenic which prompted cessation of treatment. With limited options by 3/2005, the patient underwent re-irradiation in the form of intensity-modulated radiation therapy (IMRT) given 120 cGy BID for a total of 60 Gy over 5 weeks to gross sites of disease. Treatment significantly overlapped previously irradiated sites from both his initial 3D radiation and radiosurgery treatment. When his white blood count returned to normal on 3/14/2005, concurrent chemotherapy with carboplatin and paclitaxel was initiated. Concurrent chemoradiation was completed by 4/13/05. The patient had developed dysphagia, trismus, persistent xerostomia, grade 3 mucositis, excessive mucus production, and fatigue. Post-treatment MRI 06/05 showed an overall reduction in bulk of the right nasopharyngeal mass and stable appearance of tumor extension into the right cavernous sinus, pteryopalatine fossa, and skull base.

In 08/2005, patient was admitted to inpatient oncology service for 7 days for nausea/emesis along with trismus, decreased oral intake, and resultant dehydration. Extensive scarring of the TMJ from prior tumor and radiation therapy was apparent on exam. To avoid airway compromise and improve nutrition, tracheostomy was performed, and an open gastrostomy tube was placed.

During the hospital admission, MRI showed marked progression of disease. A large necrotic mass centered in the right nasopharyngeal region, extending inferiorly to the level of the right pyriform sinus as well as anteriorly into the infratemporal fossa, was seen. There was again evidence for intracranial extension of disease (Figure 3, part G). Hospice care was recommended to the patient and his family, but the patient declined.

The patient therefore went on to receive cetuximab from 10/2005 to 3/2006, at 400 mg/m^2 ^loading dose and weekly doses of 250 mg/m^2^. After 3 treatments, he reported a marked decrease in pain and significant improvement in the range of motion in his neck. Repeat MRI 4/2006 revealed no significant disease progression (Figure 3, part H). By this time, the patient had returned to a normal activity level and had pain level ranging 0–2/10 without medication. Repeat MRI 1/07 remained stable.

Unfortunately, in 2/2007, patient was noted to have a new right-sided facial droop along with a new anaerobic smell and facial pain. He was again reinitiated on weekly cetuximab therapy, at a loading dose of 400 mg/m^2 ^followed by weekly maintenance of 250 mg/m^2^. After his first cycle, he noted improved breathing through his right nostril and decreased odor. By his third cycle, he had developed a grade 2 acneiform rash over his face, scalp, and trunk, which was treated with doxycycline 100 mg BID. He was maintained on cetuximab through 8/2007, when MRI in 8/2007 continued to reveal stable disease. Throughout his 6-month course of cetuximab, he developed significant issues with hypokalemia attributed to cetuximab. This resolved after treatment was discontinued.

The patient remained stable off any systemic therapy until 7/2008. At that time, he developed clinical local progression of disease resulting in epistaxis, increased odor, and breakdown tissue in preauricular region. MRI revealed increasing abnormal enhancement and mass involving the right nasopharyngeal cavity extending into the right cavernous sinus, middle cranial fossa, and dural surface. He was subsequently given 40 mg/m^2 ^methotrexate and clindamycin. There appeared to be an initial clinical response, with decreased erythema in the preauricular region, increased energy, and less odor. However, in 10/2008, progression of disease was demonstrated by increased sinus congestion, bleeding, and odor despite aggressive oral hygiene and antibiotic treatment. Additionally, MRI revealed tumor progression into the nasal cavity.

The patient was being considered for a phase I trial, but his tumor was rapidly progressing with worsening of his symptoms. He therefore was placed on weekly 450 mg/m^2 ^5-fluorouracil (5-FU) and 20 mg/m^2 ^leucovorin in 11/2008. Surprisingly, his symptoms have improved significantly. During treatment through 1/21/2009, he developed neutropenic fever which required hospitalization and IV antibiotics. Presently, the patient is scheduled to resume his systemic therapy.

A summary of the patient's disease and treatment course can be found in table 1.

**Table 1:** Summary and outcomes of total radiation and chemotherapy received.

Date	Time to progression	Radiation target	Type of radiation therapy	Other therapy
11/97 – 01/98	N/A	Nasopharynx	3D Conformal, 66 Gy/2.0 Gy in 33 fractions	Concurrent Cisplatin + 5 FU

02/98	4 years	Nasopharynx	Stereotactic radiosurgery, 12 Gy in 1 fraction	Adjuvant Cisplatin + 5 FU

03/02	2 years	Nasopharynx	Stereotactic radiosurgery, 12 Gy in 1 fraction	None

09/04 – 02/05, 03/05 – 04/05	12 months	Nasopharynx	IMRT, 60 Gy/1.2 Gy in 50 fractions	Concurrent Carboplatin + Taxol

10/05 – 03/06, 02/07 – 08/07	30 months	N/A	N/A	Cetuximab

07/08 – 10/08	0 months	N/A	N/A	Methotrexate

11/08 – present	TBD	N/A	N/A	5 FU + leucovorin

Total Radiation Received: 158 Gy to the nasopharynx				

## Discussion

In this case report, we present a patient with locoregionally advanced lympho-epithelial carcinoma of the nasopharynx. Nasopharyngeal carcinoma typically originates in the fossa of Rosenmüller. Since this is a clinically occult site, patients often remain asymptomatic for a relatively prolonged period. Frequently, symptoms of epistaxis, nasal congestion, or otitis media are treated for benign etiologies which defer a definitive diagnosis (mean 7.2 months in one series) [8]. Consequently, more than 90 percent of patients present with locally and/or regionally advanced disease at diagnosis, often with cranial nerve defecits and/or massive cervical adenopathy [9,10]. Additionally, a high incidence (20–40%) of distant metastases has been reported [11].

Due to anatomical limitation to surgical interventions, radiotherapy with chemotherapy is the preferred choice of treatment. The most pivotal trial investigating the use of chemoradiation in the treatment of nasopharyngeal cancer came from the Intergroup. The chemotherapy regimen consisted of cisplatin during radiation and cisplatin and 5-FU adjuvant. The 3 year progression free survival was 69% in the chemoradiation arm versus 24% with radiation alone. Overall survival at 3 years was 76% with chemoradiation versus 46% for radiation alone. Not surprisingly, there was a higher incidence of grade 3–4 leukopenia, nausea, and vomiting with addition of chemotherapy[2]. Multiple subsequent trials have yielded similar results [12-17]. These findings have substantiated chemoradiation for nasopharyngeal cancer. Additionally, the 5-year overall survival was 37% for WHO I, 55% for WHO II, and 60% for WHO III [18].

However, nasopharyngeal carcinoma still has a recurrence rate of 31% post-chemoradiation therapy at 3 years [2]. External beam radiation, in the form of intensity-modulated radiation, stereotactic radiosurgery, hyperfractionated radiation, or intracavitary brachytherapy may be options for patients who have a local or regional recurrence. Induction chemotherapy with agents such as cisplatin, taxanes, 5-fluoruracil, or gemcitabine may be considered prior to re-irradiation [19].

Stereotactic radiosurgery (SRS) uses three dimensional planning and techniques to precisely deliver narrowly collimated beams of ionizing radiation in a single high-dose fraction to small (<4 cm) targets. Administering an SRS boost following external beam RT may improve local control in locally advanced NPC. In one series, all of 23 consecutive patients with stage IV disease receiving an SRS boost following fractionated RT were locally controlled at a mean follow-up of 21 months, although eight subsequently developed regional or distant metastases [20]. Stereotactic techniques and proton beam therapy have also been used to salvage patients with locally recurrent or persistent disease. As an example, in a report of 36 patients with recurrent NPC who were re-irradiated with a radiosurgical boost, the three year locoregional control rate was 56 percent, and a five-year survival of 49 percent [21,22].

Cetuximab, a monoclonal chimeric antibody, inhibits EGFR, which is over-expressed in the vast majority of head and neck cancers [23]. Cetuximab inhibits receptor activity by blocking the ligand binding site. In addition to its potential as a radiation sensitizer [7,24], cetuximab has also been investigated as a single agent, and in combination with cytotoxic agents in patients with advanced head and neck cancer [25-28]. The benefit of cetuximab as a radiation sensitizer was tested in a multinational trial in which 424 patients with locoregionally advanced SCC of the oropharynx, hypopharynx, or larynx were randomly assigned to radiotherapy (once daily, twice daily, or with a concomitant boost, with the specific approach selected by each participating institution) with or without weekly concurrent cetuximab [7]. With a median follow-up of 54 months, the cetuximab-treated group had significantly better median (49 versus 29 months) and three-year survival (55 versus 45 percent) compared to those receiving radiation alone. Locoregional control rates were also significantly better (50 versus 41 percent), and in a separate report, there was also a suggestion of a higher laryngeal preservation rate in the cetuximab group (88 versus 80 percent at three years, respectively) [24]. The cumulative rate of distant metastases at two years was similar (16 versus 17 percent, respectively) [7].

Cetuximab has also been shown to increase locoregional disease control as well as stabilizing distant metastasis when given alone [4]. In the case of our patient, after receiving 158 Gy to the nasopharynx, additional radiotherapy was no longer deemed a viable option. Since the patient had failed multiple chemotherapy regimens including cisplatin, 5-FU, carboplatin, and paclitaxel, cetuximab was considered a reasonable choice in this patient. Anecdotally, it provided over 30 months of progression-free tumor control. Toxicities from this therapy included acneiform rash and hypokalemia.

In summary, lympho-epithelial carcinoma of the nasopharynx often exhibits a protracted course and responds to multiple treatment modalities. Up-front standard of care therapy includes concurrent cisplatin and radiation followed by adjuvant cisplatin and 5-FU. More commonly, induction chemotherapy is used for better tolerance. Despite excellent 5 year survival data with this treatment approach, a substantial proportion of patients recur, as illustrated by our patient. After exhausting multiple therapeutic strategies, EGFR inhibition afforded him over 30 months of progression-free tumor control and significant palliation.

## Consent

Verbal informed consent was obtained from the patient for publication of the case report and accompanying images.

## Competing interests

The authors declare that they have no competing interests.

## Authors' contributions

FW conceived of the study, participated in its design and coordination, and the writing of the manuscript. EY participated in the study design and coordination and writing of the manuscript. CW participated in the study design and coordination. KE participated in study design and coordination. CG participated in study design and coordination. AC participated in the study design, coordination, and writing of the manuscript. All authors read and approved the final manuscript.
